# A strategy to pioneer key agent(s) in *Cephalotaxus* alkaloids against pan-cancer via filtering methodology based on integrated pharmacology

**DOI:** 10.1186/s12967-024-05059-0

**Published:** 2024-03-10

**Authors:** Ki-Kwang Oh, Sang-Jun Yoon, Jung-A Eom, Kyeong Jin Lee, Goo-Hyun Kwon, Dong Joon Kim, Ki-Tae Suk

**Affiliations:** Institute for Liver and Digestive Diseases, College of Medicine, Hallym University, Chuncheon, 24252 Korea

**Dear Editor,**

Over the past few decades, *Cephalotaxus* alkaloids (CAs) have been considered as significant natural agents with their intriguing chemical structures and diverse bioactivities, in particular, as anti-cancer mediator. However, the investigation of its medicinal values has put in dilemma due to the limited reservoir from nature. Furthermore, the chemical synthesis of the CAs required great demanding and trial-and-error. Thus, the aim of this study was to indicate the uppermost *Cephalotaxus* alkaloid (CA) in chemical repository, via integrated data analysis.

We hypothesized the uppermost CA(s), target(s), and signaling pathway(s) can be established via cheminformatics, bioinformatics, computer screening tools, and quantum chemistry software with the holistic prospect. The CAs have potent therapeutic activities such as antileukemic, and anticancer efficacy [1]. The mainframe of structures is an azaspiranic tetracyclic scaffold (Fig. 1A). The workflow was represented in Fig. 1B.

**Fig. 1:**
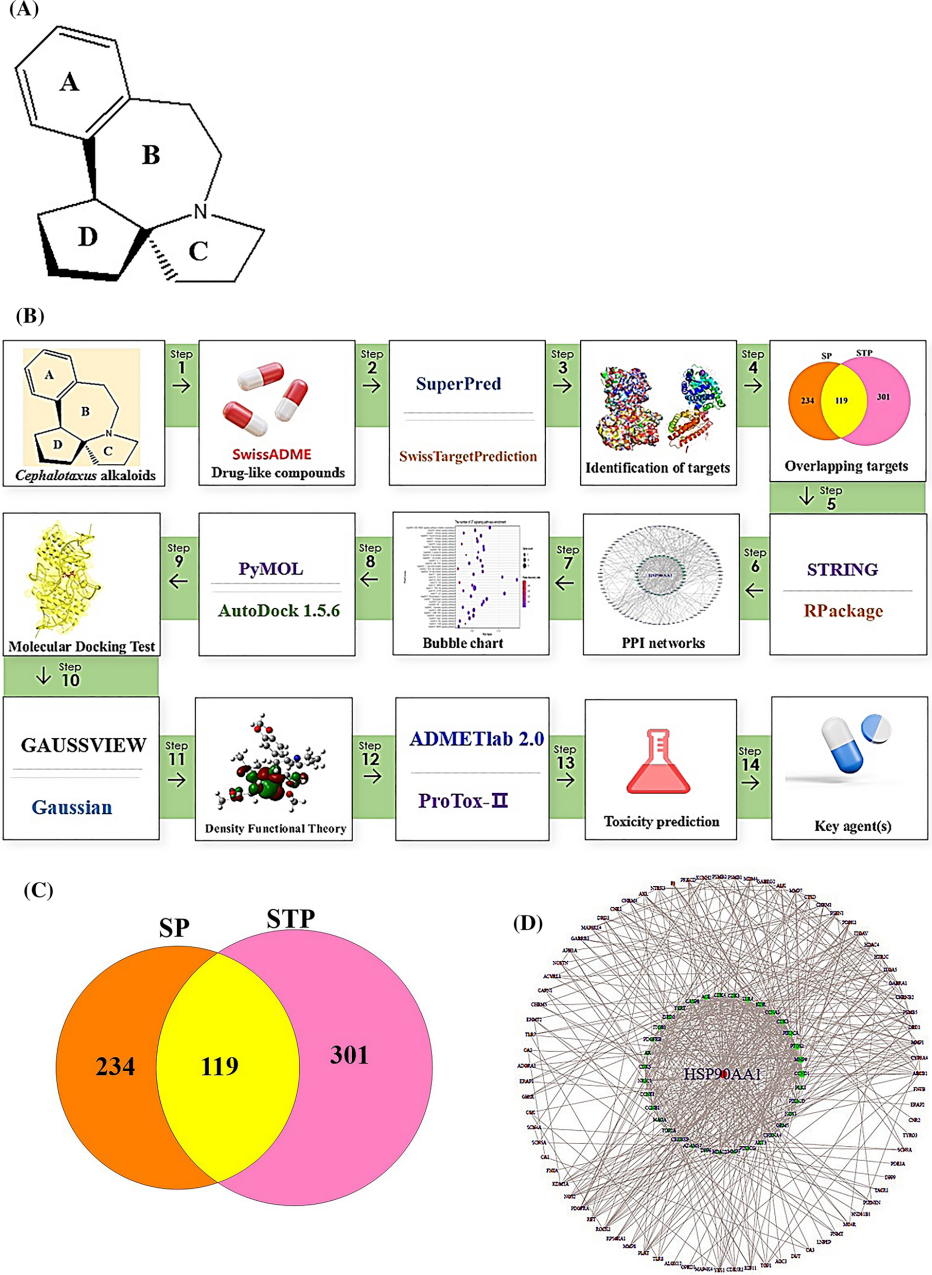

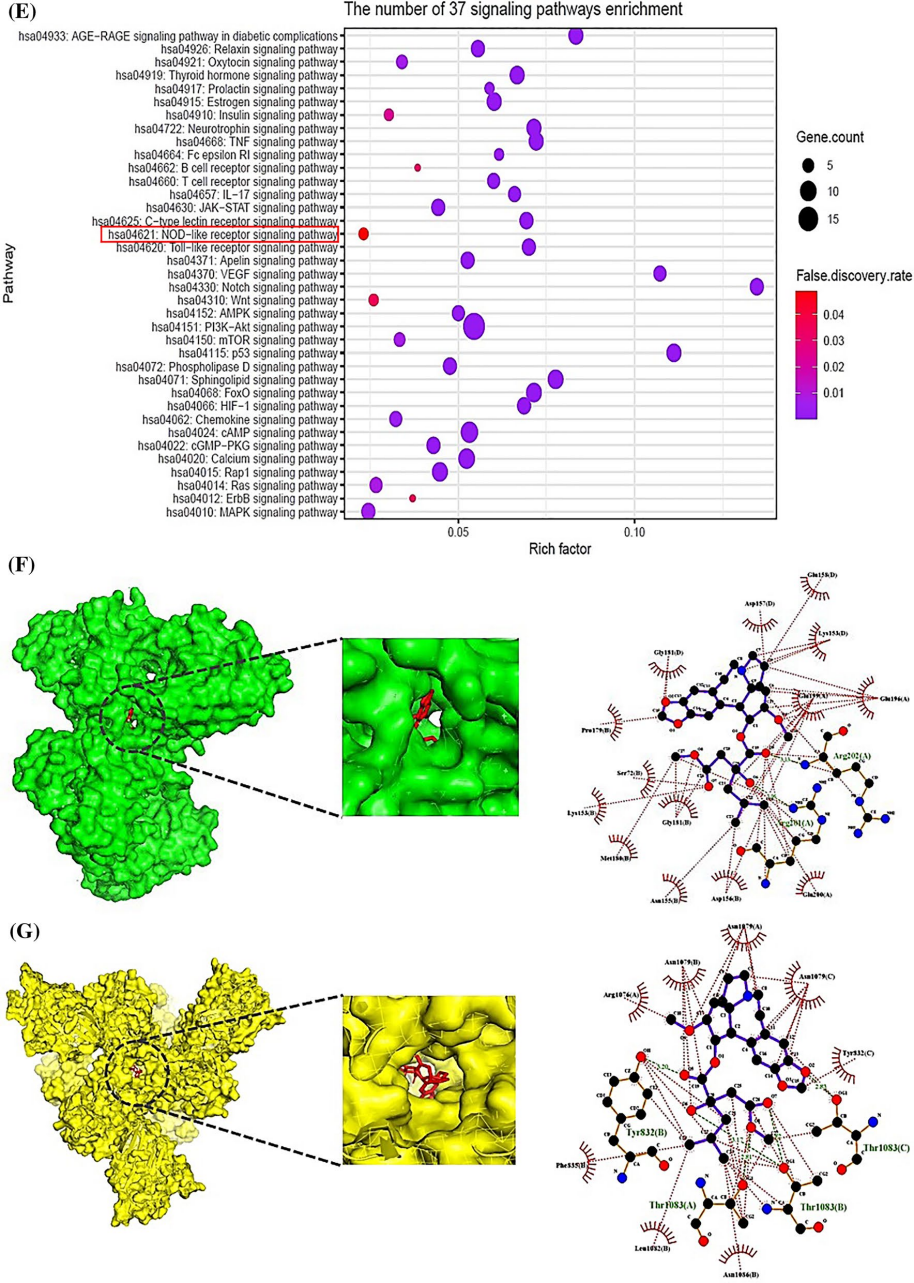

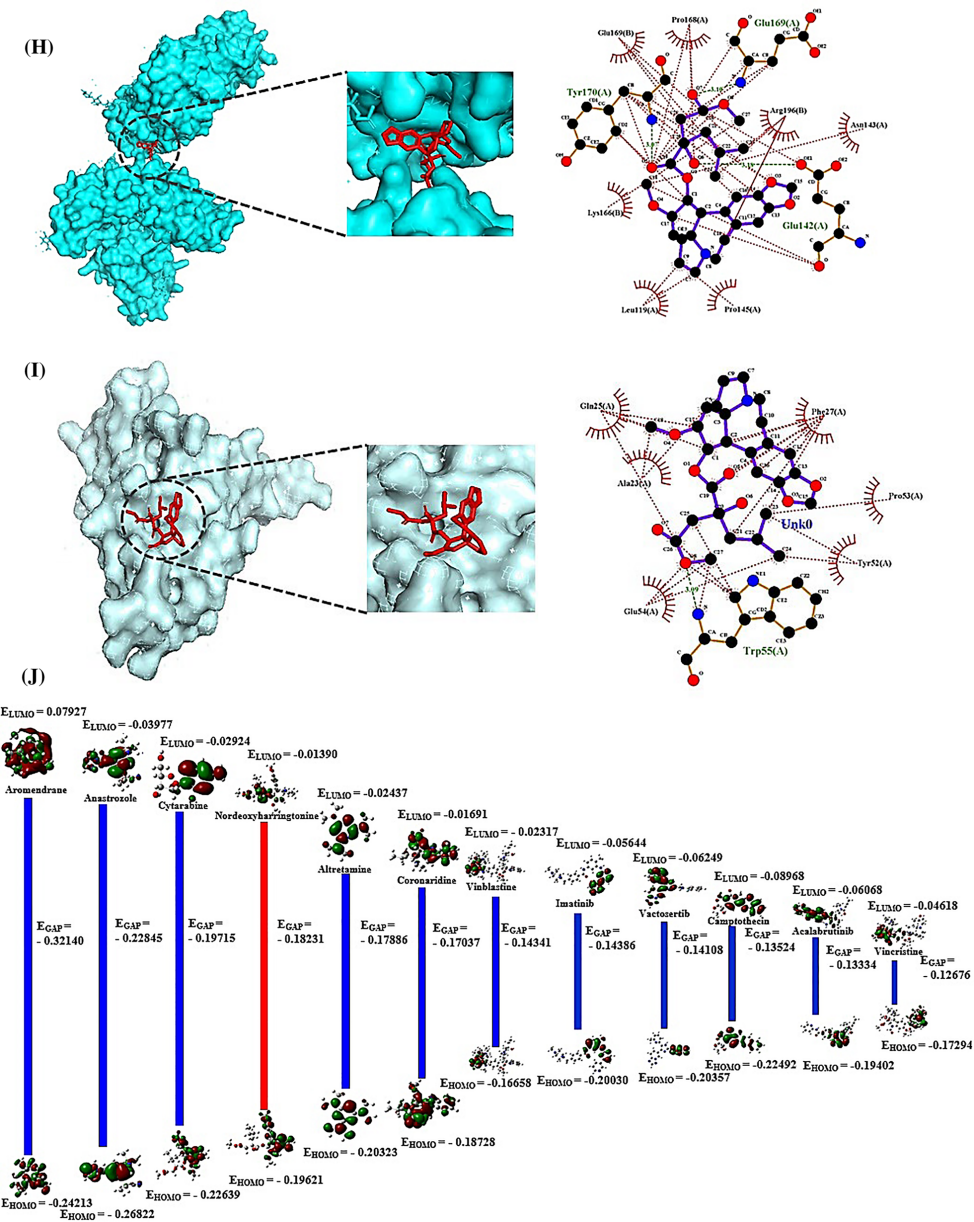

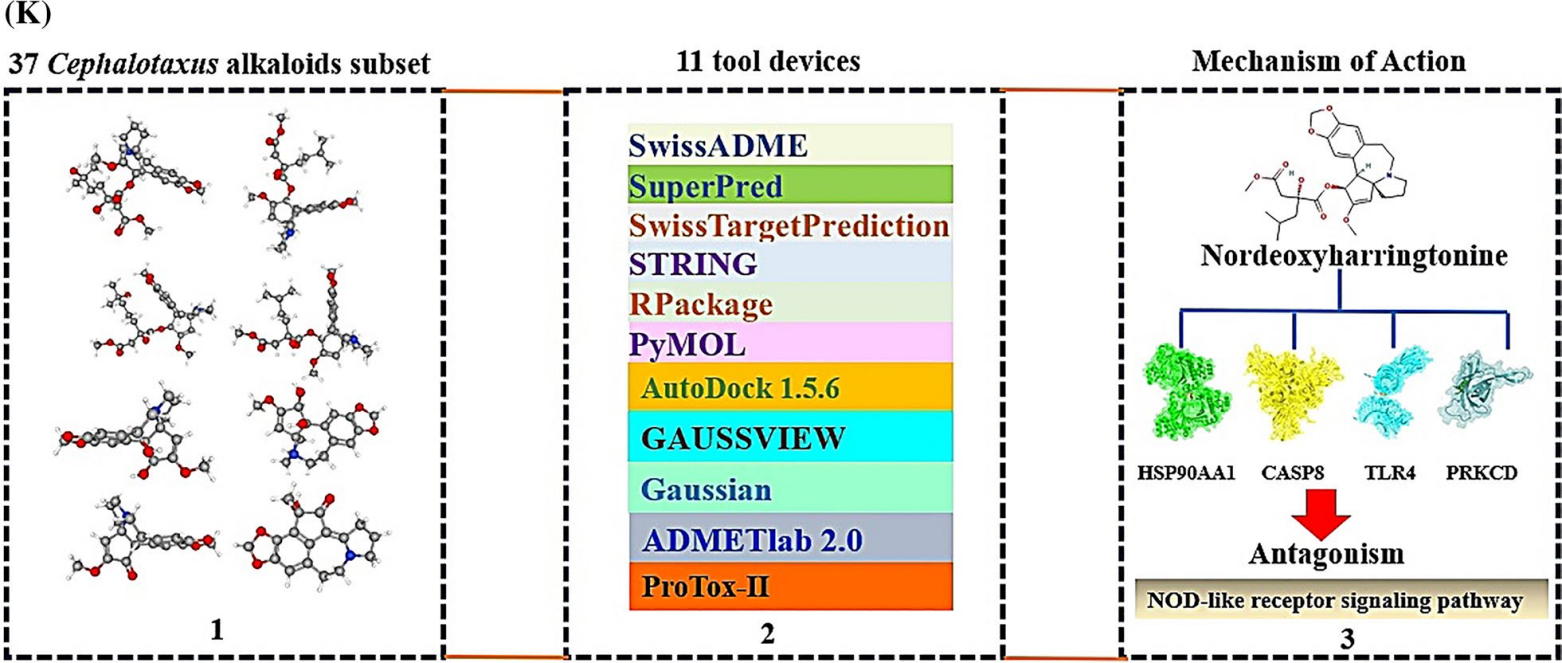
**A** The azaspiranic tetracyclic scaffold. **B** The workflow of this study. **C** The 119 overlapping targets between SP (353 targets) and STP (420 targets). **D** PPI networks (115 nodes, 603 edges). **E** A bubble chart of 37 signaling pathways against pan-cancer. **F** HSP90AA1—Nordeoxyharringtonine complex. **G** CASP8—Nordeoxyharringtonine complex. **H** TLR4—Nordeoxyharringtonine complex. **I** PRKCD—Nordeoxyharringtonine complex. **J** Density functional theory (DFT) plot and its energy gap (E_gap_) for Nordeoxyharringtonine and conventional anticancer drugs. Red bar was indicated Nordeoxyharringtonine. **K** The key summary of this study

First, the number of 37 CAs was piled by PubChem, and some literatures. The CAs were refined by Lipinski’s rule utilizing SwissADME platform, suggesting that the accepted 27 species are the key compounds for anti-cancer agents (Additional file 1: Table S1). Second, with accurate and rigor expanse, the intersecting targets (119) were selected between 353 and 420 targets obtained by SP and STP (Fig. 1C). The STRING database, and R Package were adopted to perform protein–protein interaction (PPI) networks (115 nodes, 603 edges), identifying certain target(s) with the highest connectivity. Consequently, heat shock protein 90 alpha family class A member 1 (HSP90AA1) with the greatest degree of value (DV; 48 degrees) was the uppermost protein coding gene to hamper cancer progression (Additional file 2: Table S2), (Fig. 1D). Notably, a report demonstrated that HSP90AA1 stabilizes the cancer cell, and overexpressed in leukemia and bladder cancer [2]. It implies that inhibition of HSP90AA1 might be a potential candidate against cancer. A bubble chart shows that the number of 37 signaling pathways associated with the 119 targets was related to the occurrence and progression of cancer (Additional file 3: Table S3). Of these, NOD-like receptor (NLR) signaling pathway indicated the smallest rich factor was defined as antagonism (Fig. 1E), indicating that the inhibitors of the signaling pathway might be promising agent(s) to treat cancer [3]. Third, an overlapping CA associated with the four targets was “Nordeoxyharringtonine”, which was also confirmed as a hub compound by molecular docking assessment (MDA), and density functional theory (DFT). The Nordeoxyharringtonine formed stable complex (< − 6.0 kcal/mol) [4] in all four targets via AutoDock 1.5.6. (Fig. 1F, G, H, I; Additional file 4: Table S4). To obtain the extensive confirmation, we performed the DFT analysis with eleven conventional anticancer drugs, indicating that the softness (S) value of the eleven anticancer drugs was between 15.77785 (eV) and 6.2278 (eV) (Fig. 1J). The softness (S) depends on E_GAP_ (Energy gap; Highest Occupied Molecular Orbital (HOMO)—Lowest Unoccupied Molecular Orbital (LUMO) energy gap), the molecule along the lower energy gap is defined as better reactivity level. The below mathematical set was used to establish the reactivation of leading compounds.

$$EGAP = \,HOMO - LUMO$$

$$Hardness\,\,\,\left( \eta \right) = \,\,{{\left( {LUMO - HOMO} \right)} \mathord{\left/ {\vphantom {{\left( {LUMO - HOMO} \right)} 2}} \right. \kern-0pt} 2}$$

$$Softness\,\,\left( S \right) = {1 \mathord{\left/ {\vphantom {1 \eta }} \right. \kern-0pt} \eta }$$

$$Electronegativity\,\,\left( x \right)\, = - \,{{\left( {LUMO - HOMO} \right)} \mathord{\left/ {\vphantom {{\left( {LUMO - HOMO} \right)} 2}} \right. \kern-0pt} 2}$$

Thus, Nordeoxyharringtonine with 10.92061(eV) was within the range (15.77785–6.2278 eV), which means that Nordeoxyharringtonine might be a promising agent to use as anticancer mediator (Table 1). Finally, we investigated the toxicity via ADMETlab2.0 and ProTox-II, identifying that Nordeoxyharringtonine had no noticeable obstacles to develop a new medication (Additional file 5: Table S5).

**Table 1:** The profiling of density functional theory (DFT) with eleven conventional anti-cancer drugs and Nordeoxyharringtonine

No	Anti-cancer drugs and Nordeoxyharringtonine	LUMO	HOMO	E_GAP_ (eV)	ɳ (eV)	S (eV)	χ (eV)
1	Aromendrane (*)	0.07927	− 0.24213	− 0.32140	0.16070	6.22278	− 0.16070
2	Anastrozole (*)	− 0.03977	− 0.26822	− 0.22845	0.11423	8.75465	− 0.11423
3	Cytarabine (*)	− 0.02924	− 0.22639	− 0.19715	0.09858	10.14456	− 0.09858
4	Nordeoxyharringtonine	− 0.01390	− 0.19621	− 0.18231	0.09116	10.97033	− 0.09116
5	Altretamine (*)	− 0.02441	− 0.20323	− 0.17882	0.08941	11.18443	− 0.08941
6	Coronaridine (*)	− 0.01691	− 0.18728	− 0.17037	0.08519	11.73916	− 0.08519
7	Vinblastine (*)	− 0.02317	− 0.16658	− 0.14341	0.07171	13.94603	− 0.07171
8	Imatinib (*)	− 0.05644	− 0.20030	− 0.14386	0.07193	13.90241	− 0.07193
9	Vactosertib (*)	− 0.06249	− 0.20357	− 0.14108	0.07054	14.17635	− 0.07054
10	Camptothecin (*)	− 0.08968	− 0.22492	− 0.13524	0.06762	14.78852	− 0.06762
11	Acalabrutinib (*)	− 0.06068	− 0.19402	− 0.13334	0.06667	14.99925	− 0.06667
12	Vincristine (*)	− 0.04618	− 0.17294	− 0.12676	0.06338	15.77785	− 0.06338

(*): The conventional anti-cancer drug; LUMO: Lowest Unoccupied Molecular Orbital; HOMO: Highest Occupied Molecular Orbital; ɳ: hardness; S: softness; χ: electronegativity

In this study, we have suggested that Nordeoxyharringtonine is the most significant CA against pan-cancer. The Nordeoxyharringtonine can be paved the way to validate anti-pan-cancer in CAs as inhibitors on multiple-targets (HSP90AA1, CASP8, TLR4, and PRKCD) to NLR signaling pathway. The key summary of this study was represented in Fig. 1K.

### Supplementary Information

**Additional file 1: Table S1.** The physicochemical properties of chemical constituents.

**Additional file 2: Table S2.** The degree of value in key targets.

**Additional file 3: Table S3.** The 37 signaling pathways related to occurrence and development of cancer.

**Additional file 4: Table S4.** The binding energy of four key targets on NLR signaling pathway.

**Additional file 5: Table S5.** The toxic parameters of Nordeoxyharringtonine.

## Data Availability

All data generated or analyzed during this study are included in this published article (and its Additional files).

## Abbreviations

DFT: Density functional theory
DV: Degree of value
χ: Electronegativity
E_GAP_: Energy gap
ɳ: Hardness
HSP90AA1: Heat Shock Protein 90 Alpha family class A member 1
HOMO: Highest Occupied Molecular Orbital
LUMO: Lowest Unoccupied Molecular Orbital
MDA: Molecular docking assessment
NLR: NOD-like receptor
PPI: Protein–Protein Interaction
S: Softness
SP: SuperPred
STP: Swiss Target Prediction
